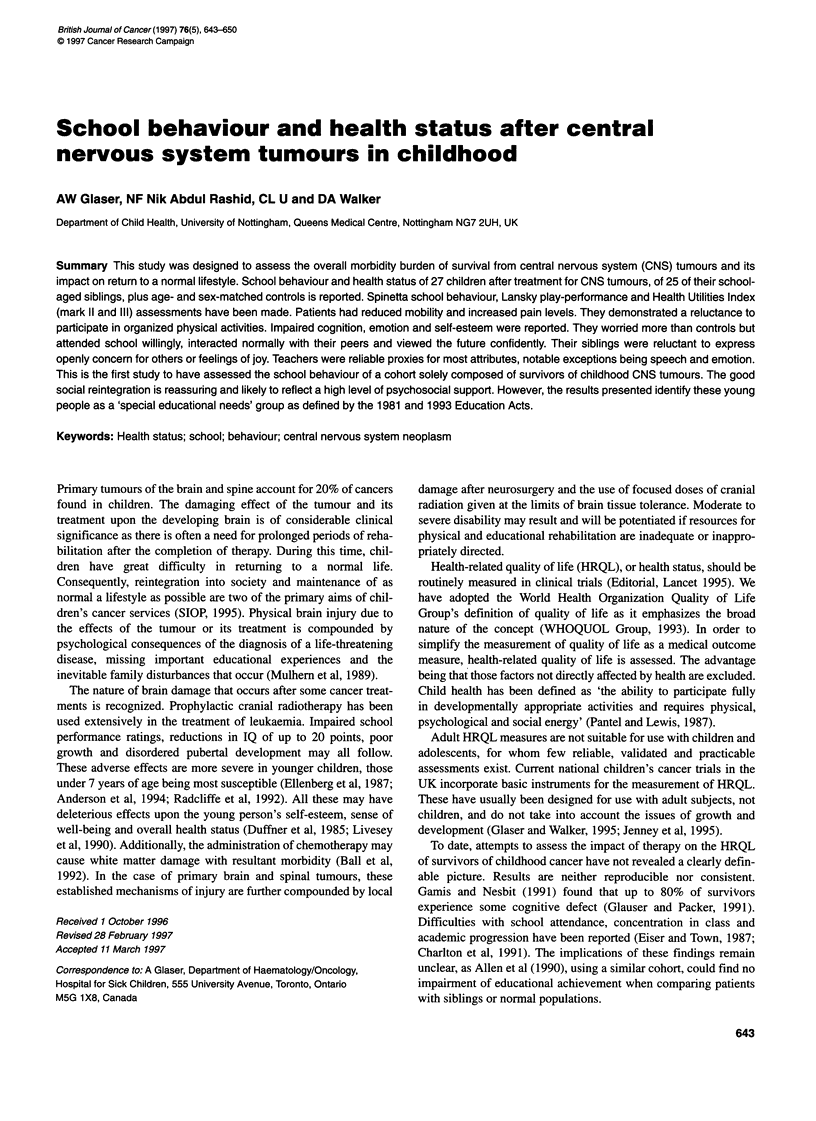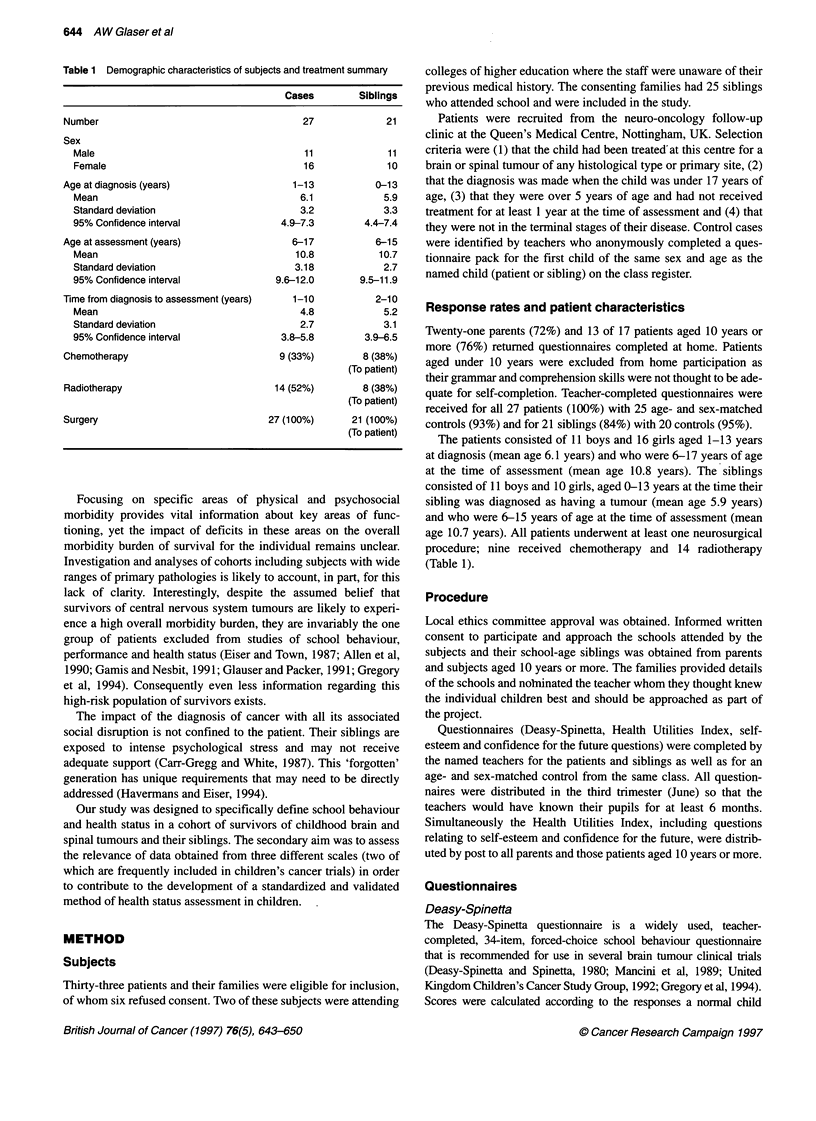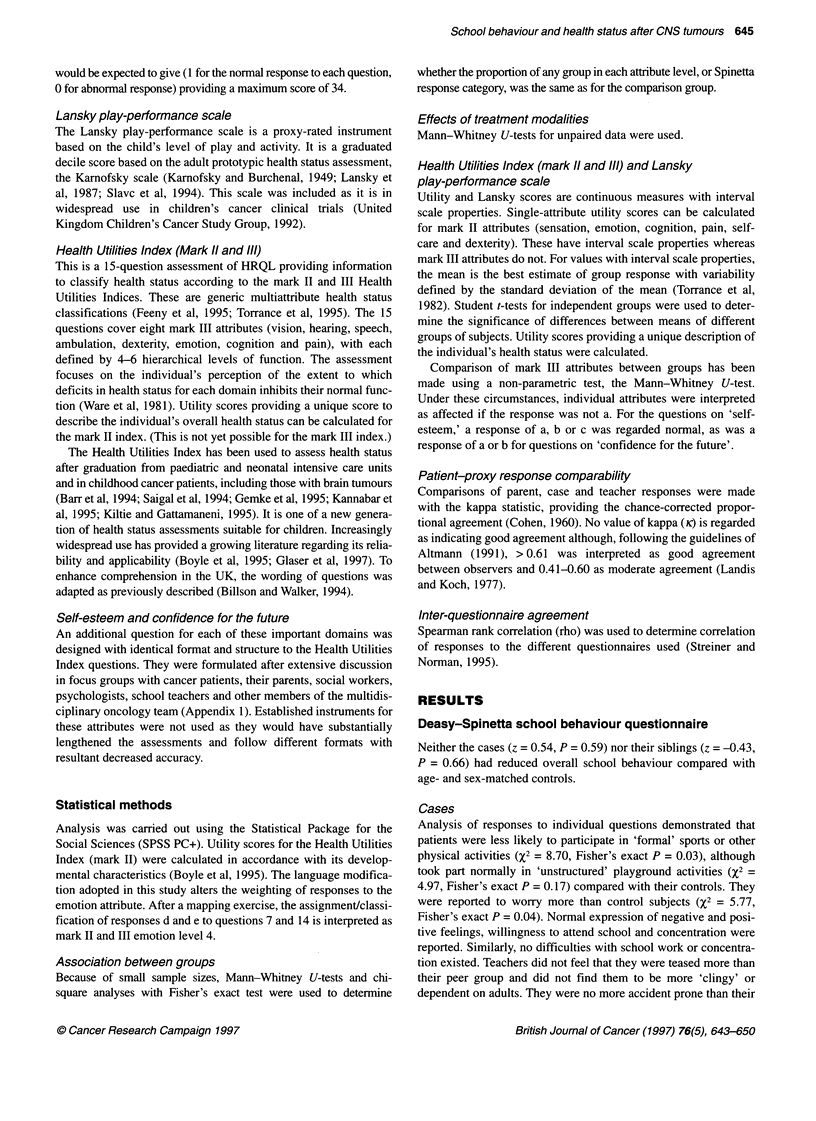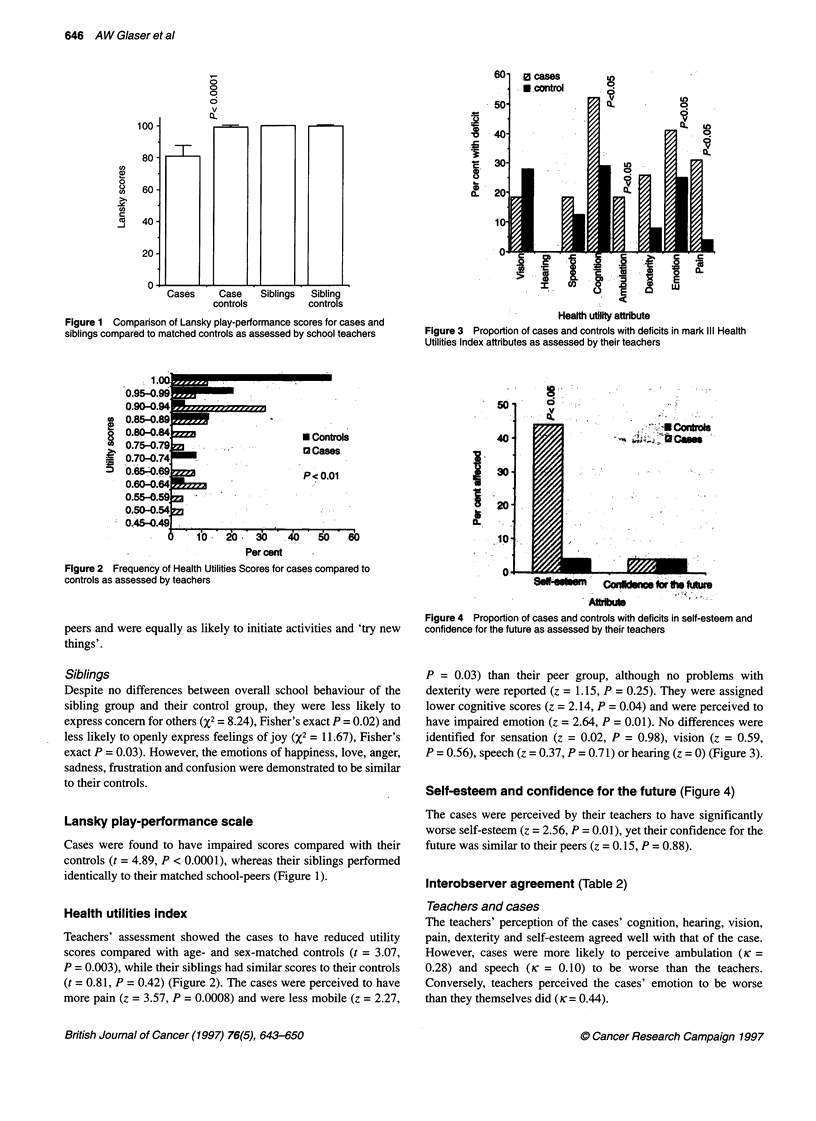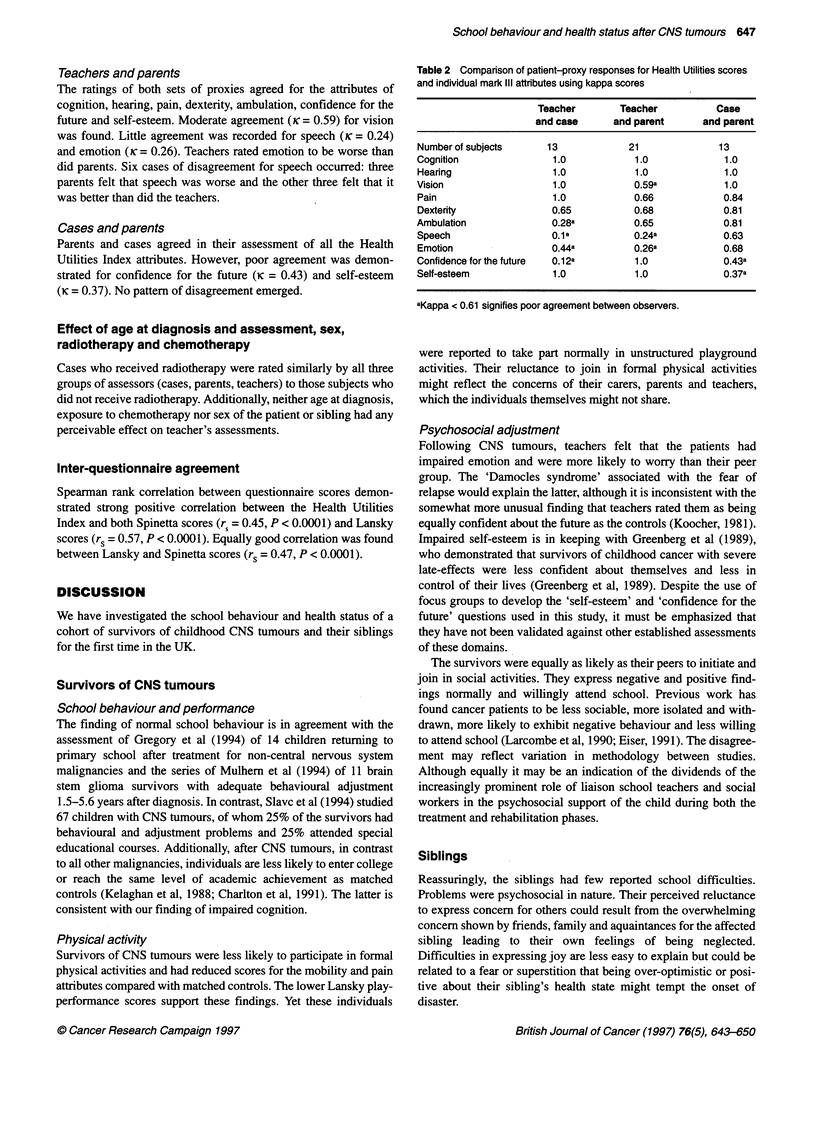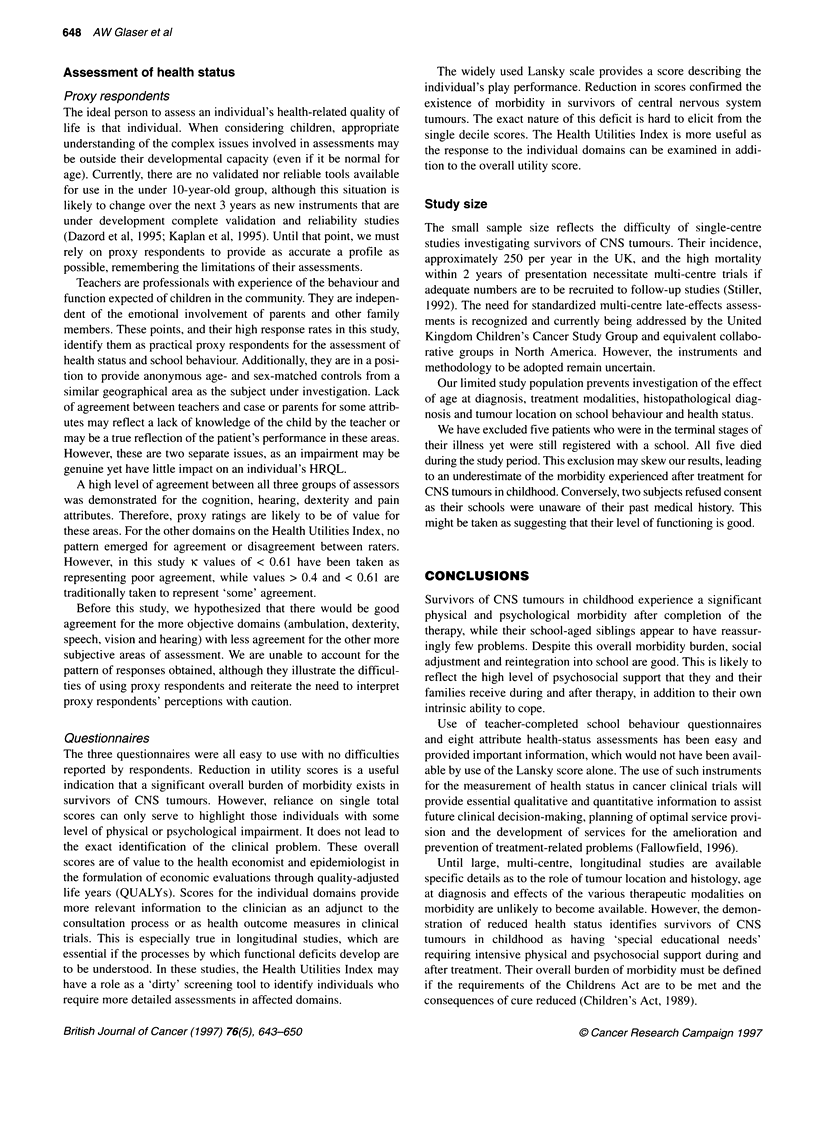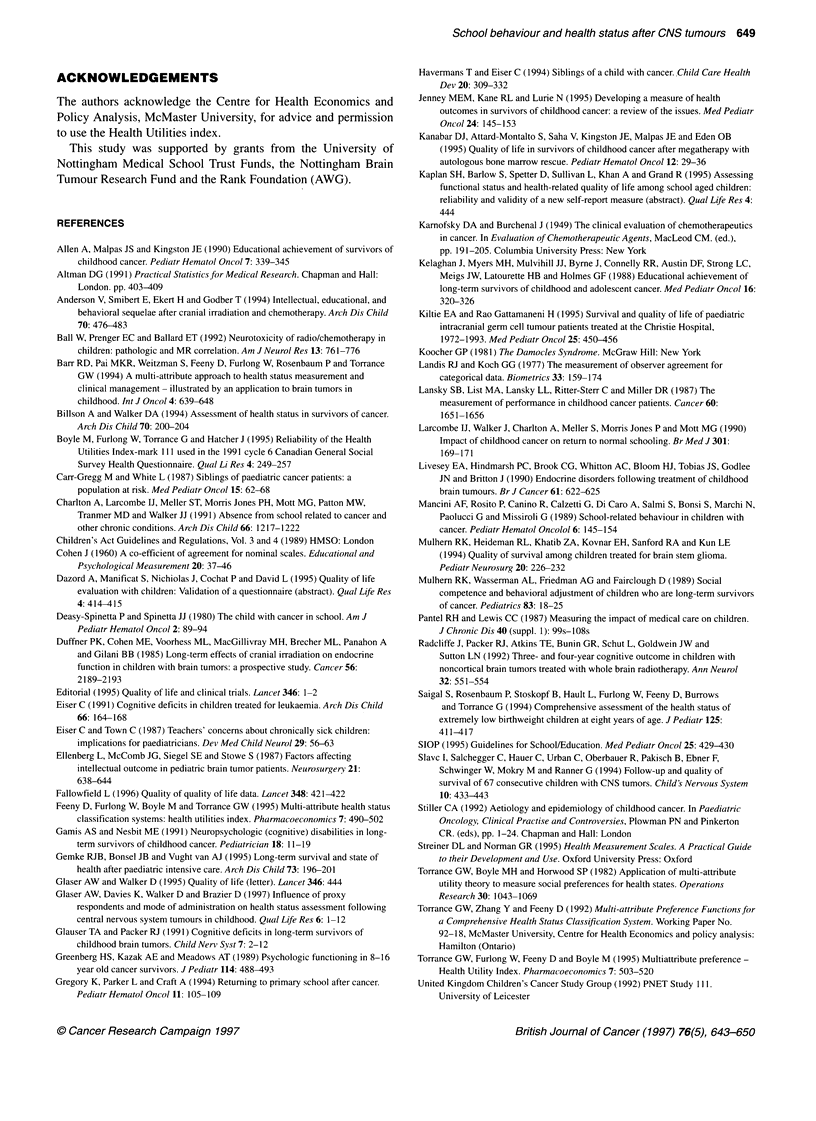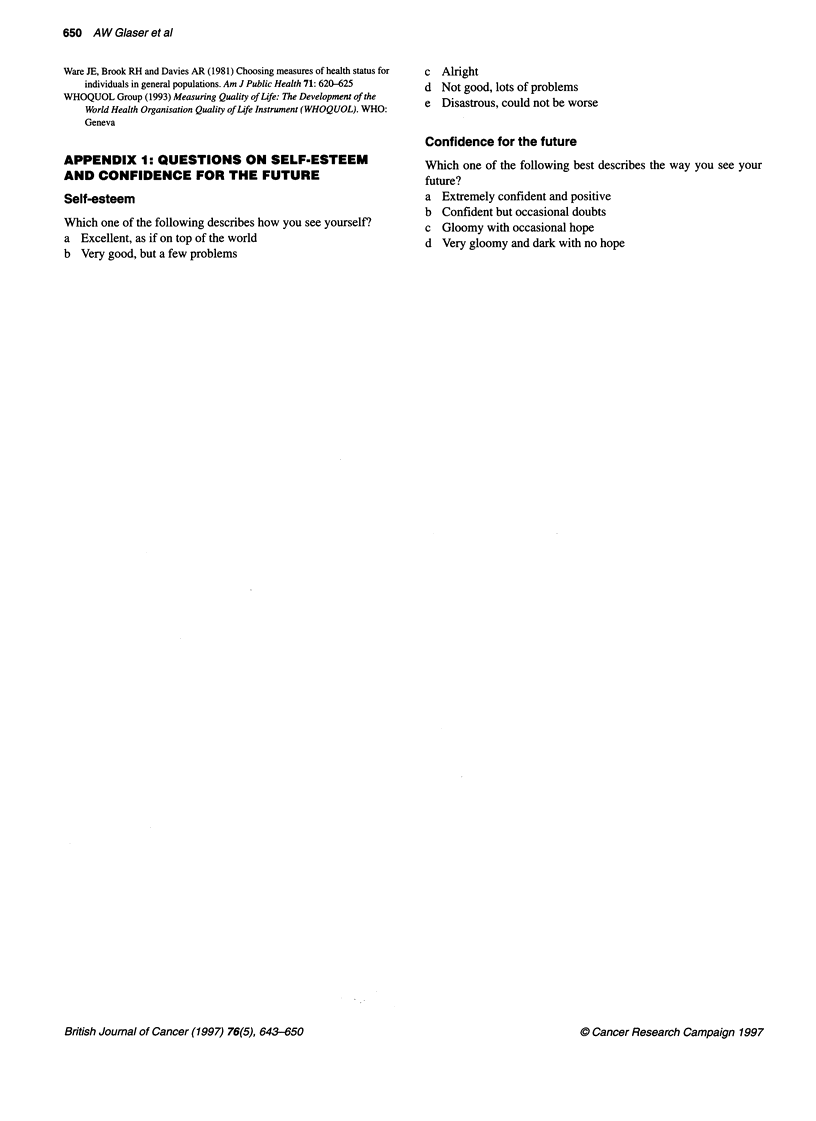# School behaviour and health status after central nervous system tumours in childhood.

**DOI:** 10.1038/bjc.1997.439

**Published:** 1997

**Authors:** A. W. Glaser, N. F. Abdul Rashid, C. L. U, D. A. Walker

**Affiliations:** Department of Child Health, University of Nottingham, Queens Medical Centre, UK.

## Abstract

This study was designed to assess the overall morbidity burden of survival from central nervous system (CNS) tumours and its impact on return to a normal lifestyle. School behaviour and health status of 27 children after treatment for CNS tumours, of 25 of their school-aged siblings, plus age- and sex-matched controls is reported. Spinetta school behaviour, Lansky play-performance and Health Utilities Index (mark II and III) assessments have been made. Patients had reduced mobility and increased pain levels. They demonstrated a reluctance to participate in organized physical activities. Impaired cognition, emotion and self-esteem were reported. They worried more than controls but attended school willingly, interacted normally with their peers and viewed the future confidently. Their siblings were reluctant to express openly concern for others or feelings of joy. Teachers were reliable proxies for most attributes, notable exceptions being speech and emotion. This is the first study to have assessed the school behaviour of a cohort solely composed of survivors of childhood CNS tumours. The good social reintegration is reassuring and likely to reflect a high level of psychosocial support. However, the results presented identify these young people as a 'special educational needs' group as defined by the 1981 and 1993 Education Acts.


British Journal of Cancer (1997) 76(5), 643–650
© 1997 Cancer Research Campaign

### School behaviour and health status after central nervous system tumours in childhood

**AW Glaser, NF Nik Abdul Rashid, CL U and DA Walker**

Department of Child Health, University of Nottingham, Queens Medical Centre, Nottingham NG7 2UH, UK

**Summary** This study was designed to assess the overall morbidity burden of survival from central nervous system (CNS) tumours and its impact on return to a normal lifestyle. School behaviour and health status of 27 children after treatment for CNS tumours, of 25 of their school-aged siblings, plus age- and sex-matched controls is reported. Spinetta school behaviour, Lansky play-performance and Health Utilities Index (mark II and III) assessments have been made. Patients had reduced mobility and increased pain levels. They demonstrated a reluctance to participate in organized physical activities. Impaired cognition, emotion and self-esteem were reported. They worried more than controls but attended school willingly, interacted normally with their peers and viewed the future confidently. Their siblings were reluctant to express openly concern for others or feelings of joy. Teachers were reliable proxies for most attributes, notable exceptions being speech and emotion. This is the first study to have assessed the school behaviour of a cohort solely composed of survivors of childhood CNS tumours. The good social reintegration is reassuring and likely to reflect a high level of psychosocial support. However, the results presented identify these young people as a 'special educational needs' group as defined by the 1981 and 1993 Education Acts.

**Keywords:** Health status; school; behaviour; central nervous system neoplasm

Primary tumours of the brain and spine account for 20% of cancers found in children. The damaging effect of the tumour and its treatment upon the developing brain is of considerable clinical significance as there is often a need for prolonged periods of rehabilitation after the completion of therapy. During this time, children have great difficulty in returning to a normal life. Consequently, reintegration into society and maintenance of as normal a lifestyle as possible are two of the primary aims of children's cancer services (SIOP, 1995). Physical brain injury due to the effects of the tumour or its treatment is compounded by psychological consequences of the diagnosis of a life-threatening disease, missing important educational experiences and the inevitable family disturbances that occur (Mulhern et al, 1989).

The nature of brain damage that occurs after some cancer treatments is recognized. Prophylactic cranial radiotherapy has been used extensively in the treatment of leukaemia. Impaired school performance ratings, reductions in IQ of up to 20 points, poor growth and disordered pubertal development may all follow. These adverse effects are more severe in younger children, those under 7 years of age being most susceptible (Ellenberg et al, 1987; Anderson et al, 1994; Radcliffe et al, 1992). All these may have deleterious effects upon the young person's self-esteem, sense of well-being and overall health status (Duffner et al, 1985; Livesey et al, 1990). Additionally, the administration of chemotherapy may cause white matter damage with resultant morbidity (Ball et al, 1992). In the case of primary brain and spinal tumours, these established mechanisms of injury are further compounded by local damage after neurosurgery and the use of focused doses of cranial radiation given at the limits of brain tissue tolerance. Moderate to severe disability may result and will be potentiated if resources for physical and educational rehabilitation are inadequate or inappropriately directed.

Health-related quality of life (HRQL), or health status, should be routinely measured in clinical trials (Editorial, Lancet 1995). We have adopted the World Health Organization Quality of Life Group's definition of quality of life as it emphasizes the broad nature of the concept (WHOQUOL Group, 1993). In order to simplify the measurement of quality of life as a medical outcome measure, health-related quality of life is assessed. The advantage being that those factors not directly affected by health are excluded. Child health has been defined as 'the ability to participate fully in developmentally appropriate activities and requires physical, psychological and social energy' (Pantel and Lewis, 1987).

Adult HRQL measures are not suitable for use with children and adolescents, for whom few reliable, validated and practicable assessments exist. Current national children's cancer trials in the UK incorporate basic instruments for the measurement of HRQL. These have usually been designed for use with adult subjects, not children, and do not take into account the issues of growth and development (Glaser and Walker, 1995; Jenney et al, 1995).

To date, attempts to assess the impact of therapy on the HRQL of survivors of childhood cancer have not revealed a clearly definable picture. Results are neither reproducible nor consistent. Gamis and Nesbit (1991) found that up to 80% of survivors experience some cognitive defect (Glauser and Packer, 1991). Difficulties with school attendance, concentration in class and academic progression have been reported (Eiser and Town, 1987; Charlton et al, 1991). The implications of these findings remain unclear, as Allen et al (1990), using a similar cohort, could find no impairment of educational achievement when comparing patients with siblings or normal populations.

*Received 1 October 1996*
*Revised 28 February 1997*
*Accepted 11 March 1997*

*Correspondence to:* A Glaser, Department of Haematology/Oncology, Hospital for Sick Children, 555 University Avenue, Toronto, Ontario M5G 1X8, Canada

Table 1 Demographic characteristics of subjects and treatment summary

| | Cases | Siblings |
|---|---|---|
| Number | 27 | 21 |
| Sex | | |
| Male | 11 | 11 |
| Female | 16 | 10 |
| Age at diagnosis (years) | 1–13 | 0–13 |
| Mean | 6.1 | 5.9 |
| Standard deviation | 3.2 | 3.3 |
| 95% Confidence interval | 4.9–7.3 | 4.4–7.4 |
| Age at assessment (years) | 6–17 | 6–15 |
| Mean | 10.8 | 10.7 |
| Standard deviation | 3.18 | 2.7 |
| 95% Confidence interval | 9.6–12.0 | 9.5–11.9 |
| Time from diagnosis to assessment (years) | 1–10 | 2–10 |
| Mean | 4.8 | 5.2 |
| Standard deviation | 2.7 | 3.1 |
| 95% Confidence interval | 3.8–5.8 | 3.9–6.5 |
| Chemotherapy | 9 (33%) | 8 (38%) (To patient) |
| Radiotherapy | 14 (52%) | 8 (38%) (To patient) |
| Surgery | 27 (100%) | 21 (100%) (To patient) |

Focusing on specific areas of physical and psychosocial morbidity provides vital information about key areas of functioning, yet the impact of deficits in these areas on the overall morbidity burden of survival for the individual remains unclear. Investigation and analyses of cohorts including subjects with wide ranges of primary pathologies is likely to account, in part, for this lack of clarity. Interestingly, despite the assumed belief that survivors of central nervous system tumours are likely to experience a high overall morbidity burden, they are invariably the one group of patients excluded from studies of school behaviour, performance and health status (Eiser and Town, 1987; Allen et al, 1990; Gamis and Nesbit, 1991; Glauser and Packer, 1991; Gregory et al, 1994). Consequently even less information regarding this high-risk population of survivors exists.

The impact of the diagnosis of cancer with all its associated social disruption is not confined to the patient. Their siblings are exposed to intense psychological stress and may not receive adequate support (Carr-Gregg and White, 1987). This 'forgotten' generation has unique requirements that may need to be directly addressed (Havermans and Eiser, 1994).

Our study was designed to specifically define school behaviour and health status in a cohort of survivors of childhood brain and spinal tumours and their siblings. The secondary aim was to assess the relevance of data obtained from three different scales (two of which are frequently included in children's cancer trials) in order to contribute to the development of a standardized and validated method of health status assessment in children.

### METHOD

### Subjects

Thirty-three patients and their families were eligible for inclusion, of whom six refused consent. Two of these subjects were attending colleges of higher education where the staff were unaware of their previous medical history. The consenting families had 25 siblings who attended school and were included in the study.

Patients were recruited from the neuro-oncology follow-up clinic at the Queen's Medical Centre, Nottingham, UK. Selection criteria were (1) that the child had been treated at this centre for a brain or spinal tumour of any histological type or primary site, (2) that the diagnosis was made when the child was under 17 years of age, (3) that they were over 5 years of age and had not received treatment for at least 1 year at the time of assessment and (4) that they were not in the terminal stages of their disease. Control cases were identified by teachers who anonymously completed a questionnaire pack for the first child of the same sex and age as the named child (patient or sibling) on the class register.

### Response rates and patient characteristics

Twenty-one parents (72%) and 13 of 17 patients aged 10 years or more (76%) returned questionnaires completed at home. Patients aged under 10 years were excluded from home participation as their grammar and comprehension skills were not thought to be adequate for self-completion. Teacher-completed questionnaires were received for all 27 patients (100%) with 25 age- and sex-matched controls (93%) and for 21 siblings (84%) with 20 controls (95%).

The patients consisted of 11 boys and 16 girls aged 1–13 years at diagnosis (mean age 6.1 years) and who were 6–17 years of age at the time of assessment (mean age 10.8 years). The siblings consisted of 11 boys and 10 girls, aged 0–13 years at the time their sibling was diagnosed as having a tumour (mean age 5.9 years) and who were 6–15 years of age at the time of assessment (mean age 10.7 years). All patients underwent at least one neurosurgical procedure; nine received chemotherapy and 14 radiotherapy (Table 1).

### Procedure

Local ethics committee approval was obtained. Informed written consent to participate and approach the schools attended by the subjects and their school-age siblings was obtained from parents and subjects aged 10 years or more. The families provided details of the schools and nominated the teacher whom they thought knew the individual children best and should be approached as part of the project.

Questionnaires (Deasy-Spinetta, Health Utilities Index, self-esteem and confidence for the future questions) were completed by the named teachers for the patients and siblings as well as for an age- and sex-matched control from the same class. All questionnaires were distributed in the third trimester (June) so that the teachers would have known their pupils for at least 6 months. Simultaneously the Health Utilities Index, including questions relating to self-esteem and confidence for the future, were distributed by post to all parents and those patients aged 10 years or more.

### Questionnaires

*Deasy-Spinetta*

The Deasy-Spinetta questionnaire is a widely used, teacher-completed, 34-item, forced-choice school behaviour questionnaire that is recommended for use in several brain tumour clinical trials (Deasy-Spinetta and Spinetta, 1980; Mancini et al, 1989; United Kingdom Children's Cancer Study Group, 1992; Gregory et al, 1994). Scores were calculated according to the responses a normal child

© Cancer Research Campaign 1997

would be expected to give (1 for the normal response to each question, 0 for abnormal response) providing a maximum score of 34.

### Lansky play-performance scale

The Lansky play-performance scale is a proxy-rated instrument based on the child's level of play and activity. It is a graduated decile score based on the adult prototypic health status assessment, the Karnofsky scale (Karnofsky and Burchenal, 1949; Lansky et al, 1987; Slavc et al, 1994). This scale was included as it is in widespread use in children's cancer clinical trials (United Kingdom Children's Cancer Study Group, 1992).

### Health Utilities Index (Mark II and III)

This is a 15-question assessment of HRQL providing information to classify health status according to the mark II and III Health Utilities Indices. These are generic multiattribute health status classifications (Feeny et al, 1995; Torrance et al, 1995). The 15 questions cover eight mark III attributes (vision, hearing, speech, ambulation, dexterity, emotion, cognition and pain), with each defined by 4–6 hierarchical levels of function. The assessment focuses on the individual's perception of the extent to which deficits in health status for each domain inhibits their normal function (Ware et al, 1981). Utility scores providing a unique score to describe the individual's overall health status can be calculated for the mark II index. (This is not yet possible for the mark III index.)

The Health Utilities Index has been used to assess health status after graduation from paediatric and neonatal intensive care units and in childhood cancer patients, including those with brain tumours (Barr et al, 1994; Saigal et al, 1994; Gemke et al, 1995; Kannabar et al, 1995; Kiltie and Gattamaneni, 1995). It is one of a new generation of health status assessments suitable for children. Increasingly widespread use has provided a growing literature regarding its reliability and applicability (Boyle et al, 1995; Glaser et al, 1997). To enhance comprehension in the UK, the wording of questions was adapted as previously described (Billson and Walker, 1994).

### Self-esteem and confidence for the future

An additional question for each of these important domains was designed with identical format and structure to the Health Utilities Index questions. They were formulated after extensive discussion in focus groups with cancer patients, their parents, social workers, psychologists, school teachers and other members of the multidisciplinary oncology team (Appendix 1). Established instruments for these attributes were not used as they would have substantially lengthened the assessments and follow different formats with resultant decreased accuracy.

### Statistical methods

Analysis was carried out using the Statistical Package for the Social Sciences (SPSS PC+). Utility scores for the Health Utilities Index (mark II) were calculated in accordance with its developmental characteristics (Boyle et al, 1995). The language modification adopted in this study alters the weighting of responses to the emotion attribute. After a mapping exercise, the assignment/classification of responses d and e to questions 7 and 14 is interpreted as mark II and III emotion level 4.

### Association between groups

Because of small sample sizes, Mann–Whitney *U*-tests and chi-square analyses with Fisher's exact test were used to determine whether the proportion of any group in each attribute level, or Spinetta response category, was the same as for the comparison group.

### Effects of treatment modalities

Mann–Whitney *U*-tests for unpaired data were used.

### Health Utilities Index (mark II and III) and Lansky play-performance scale

Utility and Lansky scores are continuous measures with interval scale properties. Single-attribute utility scores can be calculated for mark II attributes (sensation, emotion, cognition, pain, self-care and dexterity). These have interval scale properties whereas mark III attributes do not. For values with interval scale properties, the mean is the best estimate of group response with variability defined by the standard deviation of the mean (Torrance et al, 1982). Student *t*-tests for independent groups were used to determine the significance of differences between means of different groups of subjects. Utility scores providing a unique description of the individual's health status were calculated.

Comparison of mark III attributes between groups has been made using a non-parametric test, the Mann–Whitney *U*-test. Under these circumstances, individual attributes were interpreted as affected if the response was not a. For the questions on 'self-esteem,' a response of a, b or c was regarded normal, as was a response of a or b for questions on 'confidence for the future'.

### Patient–proxy response comparability

Comparisons of parent, case and teacher responses were made with the kappa statistic, providing the chance-corrected proportional agreement (Cohen, 1960). No value of kappa ($\kappa$) is regarded as indicating good agreement although, following the guidelines of Altmann (1991), $> 0.61$ was interpreted as good agreement between observers and 0.41–0.60 as moderate agreement (Landis and Koch, 1977).

### Inter-questionnaire agreement

Spearman rank correlation (rho) was used to determine correlation of responses to the different questionnaires used (Streiner and Norman, 1995).

### RESULTS

### Deasy–Spinetta school behaviour questionnaire

Neither the cases ($z = 0.54$, $P = 0.59$) nor their siblings ($z = -0.43$, $P = 0.66$) had reduced overall school behaviour compared with age- and sex-matched controls.

### Cases

Analysis of responses to individual questions demonstrated that patients were less likely to participate in 'formal' sports or other physical activities ($\chi^2 = 8.70$, Fisher's exact $P = 0.03$), although took part normally in 'unstructured' playground activities ($\chi^2 = 4.97$, Fisher's exact $P = 0.17$) compared with their controls. They were reported to worry more than control subjects ($\chi^2 = 5.77$, Fisher's exact $P = 0.04$). Normal expression of negative and positive feelings, willingness to attend school and concentration were reported. Similarly, no difficulties with school work or concentration existed. Teachers did not feel that they were teased more than their peer group and did not find them to be more 'clingy' or dependent on adults. They were no more accident prone than their

© Cancer Research Campaign 1997

**Figure 1** Comparison of Lansky play-performance scores for cases and siblings compared to matched controls as assessed by school teachers

**Figure 2** Frequency of Health Utilities Scores for cases compared to controls as assessed by teachers peers and were equally as likely to initiate activities and 'try new things'.

### *Siblings*

Despite no differences between overall school behaviour of the sibling group and their control group, they were less likely to express concern for others ($\chi^2 = 8.24$), Fisher's exact $P = 0.02$) and less likely to openly express feelings of joy ($\chi^2 = 11.67$), Fisher's exact $P = 0.03$). However, the emotions of happiness, love, anger, sadness, frustration and confusion were demonstrated to be similar to their controls.

### Lansky play-performance scale

Cases were found to have impaired scores compared with their controls ($t = 4.89$, $P < 0.0001$), whereas their siblings performed identically to their matched school-peers (Figure 1).

### Health utilities index

Teachers' assessment showed the cases to have reduced utility scores compared with age- and sex-matched controls ($t = 3.07$, $P = 0.003$), while their siblings had similar scores to their controls ($t = 0.81$, $P = 0.42$) (Figure 2). The cases were perceived to have more pain ($z = 3.57$, $P = 0.0008$) and were less mobile ($z = 2.27$,

**Figure 3** Proportion of cases and controls with deficits in mark III Health Utilities Index attributes as assessed by their teachers

**Figure 4** Proportion of cases and controls with deficits in self-esteem and confidence for the future as assessed by their teachers

$P = 0.03$) than their peer group, although no problems with dexterity were reported ($z = 1.15$, $P = 0.25$). They were assigned lower cognitive scores ($z = 2.14$, $P = 0.04$) and were perceived to have impaired emotion ($z = 2.64$, $P = 0.01$). No differences were identified for sensation ($z = 0.02$, $P = 0.98$), vision ($z = 0.59$, $P = 0.56$), speech ($z = 0.37$, $P = 0.71$) or hearing ($z = 0$) (Figure 3).

### Self-esteem and confidence for the future (Figure 4)

The cases were perceived by their teachers to have significantly worse self-esteem ($z = 2.56$, $P = 0.01$), yet their confidence for the future was similar to their peers ($z = 0.15$, $P = 0.88$).

### Interobserver agreement (Table 2)

### *Teachers and cases*

The teachers' perception of the cases' cognition, hearing, vision, pain, dexterity and self-esteem agreed well with that of the case. However, cases were more likely to perceive ambulation ($\kappa = 0.28$) and speech ($\kappa = 0.10$) to be worse than the teachers. Conversely, teachers perceived the cases' emotion to be worse than they themselves did ($\kappa = 0.44$).

© Cancer Research Campaign 1997

### *Teachers and parents*

The ratings of both sets of proxies agreed for the attributes of cognition, hearing, pain, dexterity, ambulation, confidence for the future and self-esteem. Moderate agreement ($\kappa = 0.59$) for vision was found. Little agreement was recorded for speech ($\kappa = 0.24$) and emotion ($\kappa = 0.26$). Teachers rated emotion to be worse than did parents. Six cases of disagreement for speech occurred: three parents felt that speech was worse and the other three felt that it was better than did the teachers.

### *Cases and parents*

Parents and cases agreed in their assessment of all the Health Utilities Index attributes. However, poor agreement was demonstrated for confidence for the future ($\kappa = 0.43$) and self-esteem ($\kappa = 0.37$). No pattern of disagreement emerged.

### Effect of age at diagnosis and assessment, sex, radiotherapy and chemotherapy

Cases who received radiotherapy were rated similarly by all three groups of assessors (cases, parents, teachers) to those subjects who did not receive radiotherapy. Additionally, neither age at diagnosis, exposure to chemotherapy nor sex of the patient or sibling had any perceivable effect on teacher's assessments.

### Inter-questionnaire agreement

Spearman rank correlation between questionnaire scores demonstrated strong positive correlation between the Health Utilities Index and both Spinetta scores ($r_s = 0.45$, $P < 0.0001$) and Lansky scores ($r_S = 0.57$, $P < 0.0001$). Equally good correlation was found between Lansky and Spinetta scores ($r_S = 0.47$, $P < 0.0001$).

**Table 2** Comparison of patient–proxy responses for Health Utilities scores and individual mark III attributes using kappa scores

| | Teacher and case | Teacher and parent | Case and parent |
|---|---|---|---|
| Number of subjects | 13 | 21 | 13 |
| Cognition | 1.0 | 1.0 | 1.0 |
| Hearing | 1.0 | 1.0 | 1.0 |
| Vision | 1.0 | 0.59[a] | 1.0 |
| Pain | 1.0 | 0.66 | 0.84 |
| Dexterity | 0.65 | 0.68 | 0.81 |
| Ambulation | 0.28[a] | 0.65 | 0.81 |
| Speech | 0.1[a] | 0.24[a] | 0.63 |
| Emotion | 0.44[a] | 0.26[a] | 0.68 |
| Confidence for the future | 0.12[a] | 1.0 | 0.43[a] |
| Self-esteem | 1.0 | 1.0 | 0.37[a] |

[a]Kappa < 0.61 signifies poor agreement between observers.

### DISCUSSION

We have investigated the school behaviour and health status of a cohort of survivors of childhood CNS tumours and their siblings for the first time in the UK.

### Survivors of CNS tumours

### *School behaviour and performance*

The finding of normal school behaviour is in agreement with the assessment of Gregory et al (1994) of 14 children returning to primary school after treatment for non-central nervous system malignancies and the series of Mulhern et al (1994) of 11 brain stem glioma survivors with adequate behavioural adjustment 1.5–5.6 years after diagnosis. In contrast, Slavc et al (1994) studied 67 children with CNS tumours, of whom 25% of the survivors had behavioural and adjustment problems and 25% attended special educational courses. Additionally, after CNS tumours, in contrast to all other malignancies, individuals are less likely to enter college or reach the same level of academic achievement as matched controls (Kelaghan et al, 1988; Charlton et al, 1991). The latter is consistent with our finding of impaired cognition.

### *Physical activity*

Survivors of CNS tumours were less likely to participate in formal physical activities and had reduced scores for the mobility and pain attributes compared with matched controls. The lower Lansky play-performance scores support these findings. Yet these individuals were reported to take part normally in unstructured playground activities. Their reluctance to join in formal physical activities might reflect the concerns of their carers, parents and teachers, which the individuals themselves might not share.

### *Psychosocial adjustment*

Following CNS tumours, teachers felt that the patients had impaired emotion and were more likely to worry than their peer group. The 'Damocles syndrome' associated with the fear of relapse would explain the latter, although it is inconsistent with the somewhat more unusual finding that teachers rated them as being equally confident about the future as the controls (Koocher, 1981). Impaired self-esteem is in keeping with Greenberg et al (1989), who demonstrated that survivors of childhood cancer with severe late-effects were less confident about themselves and less in control of their lives (Greenberg et al, 1989). Despite the use of focus groups to develop the 'self-esteem' and 'confidence for the future' questions used in this study, it must be emphasized that they have not been validated against other established assessments of these domains.

The survivors were equally as likely as their peers to initiate and join in social activities. They express negative and positive findings normally and willingly attend school. Previous work has found cancer patients to be less sociable, more isolated and withdrawn, more likely to exhibit negative behaviour and less willing to attend school (Larcombe et al, 1990; Eiser, 1991). The disagreement may reflect variation in methodology between studies. Although equally it may be an indication of the dividends of the increasingly prominent role of liaison school teachers and social workers in the psychosocial support of the child during both the treatment and rehabilitation phases.

### Siblings

Reassuringly, the siblings had few reported school difficulties. Problems were psychosocial in nature. Their perceived reluctance to express concern for others could result from the overwhelming concern shown by friends, family and aquaintances for the affected sibling leading to their own feelings of being neglected. Difficulties in expressing joy are less easy to explain but could be related to a fear or superstition that being over-optimistic or positive about their sibling's health state might tempt the onset of disaster.

© Cancer Research Campaign 1997

### Assessment of health status

### *Proxy respondents*

The ideal person to assess an individual's health-related quality of life is that individual. When considering children, appropriate understanding of the complex issues involved in assessments may be outside their developmental capacity (even if it be normal for age). Currently, there are no validated nor reliable tools available for use in the under 10-year-old group, although this situation is likely to change over the next 3 years as new instruments that are under development complete validation and reliability studies (Dazord et al, 1995; Kaplan et al, 1995). Until that point, we must rely on proxy respondents to provide as accurate a profile as possible, remembering the limitations of their assessments.

Teachers are professionals with experience of the behaviour and function expected of children in the community. They are independent of the emotional involvement of parents and other family members. These points, and their high response rates in this study, identify them as practical proxy respondents for the assessment of health status and school behaviour. Additionally, they are in a position to provide anonymous age- and sex-matched controls from a similar geographical area as the subject under investigation. Lack of agreement between teachers and case or parents for some attributes may reflect a lack of knowledge of the child by the teacher or may be a true reflection of the patient's performance in these areas. However, these are two separate issues, as an impairment may be genuine yet have little impact on an individual's HRQL.

A high level of agreement between all three groups of assessors was demonstrated for the cognition, hearing, dexterity and pain attributes. Therefore, proxy ratings are likely to be of value for these areas. For the other domains on the Health Utilities Index, no pattern emerged for agreement or disagreement between raters. However, in this study $\kappa$ values of $< 0.61$ have been taken as representing poor agreement, while values $> 0.4$ and $< 0.61$ are traditionally taken to represent 'some' agreement.

Before this study, we hypothesized that there would be good agreement for the more objective domains (ambulation, dexterity, speech, vision and hearing) with less agreement for the other more subjective areas of assessment. We are unable to account for the pattern of responses obtained, although they illustrate the difficulties of using proxy respondents and reiterate the need to interpret proxy respondents' perceptions with caution.

### *Questionnaires*

The three questionnaires were all easy to use with no difficulties reported by respondents. Reduction in utility scores is a useful indication that a significant overall burden of morbidity exists in survivors of CNS tumours. However, reliance on single total scores can only serve to highlight those individuals with some level of physical or psychological impairment. It does not lead to the exact identification of the clinical problem. These overall scores are of value to the health economist and epidemiologist in the formulation of economic evaluations through quality-adjusted life years (QUALYs). Scores for the individual domains provide more relevant information to the clinician as an adjunct to the consultation process or as health outcome measures in clinical trials. This is especially true in longitudinal studies, which are essential if the processes by which functional deficits develop are to be understood. In these studies, the Health Utilities Index may have a role as a 'dirty' screening tool to identify individuals who require more detailed assessments in affected domains.

The widely used Lansky scale provides a score describing the individual's play performance. Reduction in scores confirmed the existence of morbidity in survivors of central nervous system tumours. The exact nature of this deficit is hard to elicit from the single decile scores. The Health Utilities Index is more useful as the response to the individual domains can be examined in addition to the overall utility score.

### Study size

The small sample size reflects the difficulty of single-centre studies investigating survivors of CNS tumours. Their incidence, approximately 250 per year in the UK, and the high mortality within 2 years of presentation necessitate multi-centre trials if adequate numbers are to be recruited to follow-up studies (Stiller, 1992). The need for standardized multi-centre late-effects assessments is recognized and currently being addressed by the United Kingdom Children's Cancer Study Group and equivalent collaborative groups in North America. However, the instruments and methodology to be adopted remain uncertain.

Our limited study population prevents investigation of the effect of age at diagnosis, treatment modalities, histopathological diagnosis and tumour location on school behaviour and health status.

We have excluded five patients who were in the terminal stages of their illness yet were still registered with a school. All five died during the study period. This exclusion may skew our results, leading to an underestimate of the morbidity experienced after treatment for CNS tumours in childhood. Conversely, two subjects refused consent as their schools were unaware of their past medical history. This might be taken as suggesting that their level of functioning is good.

### CONCLUSIONS

Survivors of CNS tumours in childhood experience a significant physical and psychological morbidity after completion of the therapy, while their school-aged siblings appear to have reassuringly few problems. Despite this overall morbidity burden, social adjustment and reintegration into school are good. This is likely to reflect the high level of psychosocial support that they and their families receive during and after therapy, in addition to their own intrinsic ability to cope.

Use of teacher-completed school behaviour questionnaires and eight attribute health-status assessments has been easy and provided important information, which would not have been available by use of the Lansky score alone. The use of such instruments for the measurement of health status in cancer clinical trials will provide essential qualitative and quantitative information to assist future clinical decision-making, planning of optimal service provision and the development of services for the amelioration and prevention of treatment-related problems (Fallowfield, 1996).

Until large, multi-centre, longitudinal studies are available specific details as to the role of tumour location and histology, age at diagnosis and effects of the various therapeutic modalities on morbidity are unlikely to become available. However, the demonstration of reduced health status identifies survivors of CNS tumours in childhood as having 'special educational needs' requiring intensive physical and psychosocial support during and after treatment. Their overall burden of morbidity must be defined if the requirements of the Childrens Act are to be met and the consequences of cure reduced (Children's Act, 1989).

© Cancer Research Campaign 1997

### ACKNOWLEDGEMENTS

The authors acknowledge the Centre for Health Economics and Policy Analysis, McMaster University, for advice and permission to use the Health Utilities index.

This study was supported by grants from the University of Nottingham Medical School Trust Funds, the Nottingham Brain Tumour Research Fund and the Rank Foundation (AWG).

### REFERENCES

Allen A, Malpas JS and Kingston JE (1990) Educational achievement of survivors of childhood cancer. *Pediatr Hematol Oncol* **7**: 339–345

Altman DG (1991) *Practical Statistics for Medical Research*. Chapman and Hall: London. pp. 403–409

Anderson V, Smibert E, Ekert H and Godber T (1994) Intellectual, educational, and behavioral sequelae after cranial irradiation and chemotherapy. *Arch Dis Child* **70**: 476–483

Ball W, Prenger EC and Ballard ET (1992) Neurotoxicity of radio/chemotherapy in children: pathologic and MR correlation. *Am J Neurol Res* **13**: 761–776

Barr RD, Pai MKR, Weitzman S, Feeny D, Furlong W, Rosenbaum P and Torrance GW (1994) A multi-attribute approach to health status measurement and clinical management – illustrated by an application to brain tumors in childhood. *Int J Oncol* **4**: 639–648

Billson A and Walker DA (1994) Assessment of health status in survivors of cancer. *Arch Dis Child* **70**: 200–204

Boyle M, Furlong W, Torrance G and Hatcher J (1995) Reliability of the Health Utilities Index-mark 111 used in the 1991 cycle 6 Canadian General Social Survey Health Questionnaire. *Qual Li Res* **4**: 249–257

Carr-Gregg M and White L (1987) Siblings of paediatric cancer patients: a population at risk. *Med Pediatr Oncol* **15**: 62–68

Charlton A, Larcombe IJ, Meller ST, Morris Jones PH, Mott MG, Patton MW, Tranmer MD and Walker JJ (1991) Absence from school related to cancer and other chronic conditions. *Arch Dis Child* **66**: 1217–1222

Children's Act Guidelines and Regulations, Vol. 3 and 4 (1989) HMSO: London

Cohen J (1960) A co-efficient of agreement for nominal scales. *Educational and Psychological Measurement* **20**: 37–46

Dazord A, Manificat S, Nichiolas J, Cochat P and David L (1995) Quality of life evaluation with children: Validation of a questionnaire (abstract). *Qual Life Res* **4**: 414–415

Deasy-Spinetta P and Spinetta JJ (1980) The child with cancer in school. *Am J Pediatr Hematol Oncol* **2**: 89–94

Duffner PK, Cohen ME, Voorhess ML, MacGillivray MH, Brecher ML, Panahon A and Gilani BB (1985) Long-term effects of cranial irradiation on endocrine function in children with brain tumors: a prospective study. *Cancer* **56**: 2189–2193

Editorial (1995) Quality of life and clinical trials. *Lancet* **346**: 1–2

Eiser C (1991) Cognitive deficits in children treated for leukaemia. *Arch Dis Child* **66**: 164–168

Eiser C and Town C (1987) Teachers' concerns about chronically sick children: implications for paediatricians. *Dev Med Child Neurol* **29**: 56–63

Ellenberg L, McComb JG, Siegel SE and Stowe S (1987) Factors affecting intellectual outcome in pediatric brain tumor patients. *Neurosurgery* **21**: 638–644

Fallowfield L (1996) Quality of quality of life data. *Lancet* **348**: 421–422

Feeny D, Furlong W, Boyle M and Torrance GW (1995) Multi-attribute health status classification systems: health utilities index. *Pharmacoeconomics* **7**: 490–502

Gamis AS and Nesbit ME (1991) Neuropsychologic (cognitive) disabilities in long-term survivors of childhood cancer. *Pediatrician* **18**: 11–19

Gemke RJB, Bonsel JB and Vught van AJ (1995) Long-term survival and state of health after paediatric intensive care. *Arch Dis Child* **73**: 196–201

Glaser AW and Walker D (1995) Quality of life (letter). *Lancet* **346**: 444

Glaser AW, Davies K, Walker D and Brazier D (1997) Influence of proxy respondents and mode of administration on health status assessment following central nervous system tumours in childhood. *Qual Life Res* **6**: 1–12

Glauser TA and Packer RJ (1991) Cognitive deficits in long-term survivors of childhood brain tumors. *Child Nerv Syst* **7**: 2–12

Greenberg HS, Kazak AE and Meadows AT (1989) Psychologic functioning in 8–16 year old cancer survivors. *J Pediatr* **114**: 488–493

Gregory K, Parker L and Craft A (1994) Returning to primary school after cancer. *Pediatr Hematol Oncol* **11**: 105–109

Havermans T and Eiser C (1994) Siblings of a child with cancer. *Child Care Health Dev* **20**: 309–332

Jenney MEM, Kane RL and Lurie N (1995) Developing a measure of health outcomes in survivors of childhood cancer: a review of the issues. *Med Pediatr Oncol* **24**: 145–153

Kanabar DJ, Attard-Montalto S, Saha V, Kingston JE, Malpas JE and Eden OB (1995) Quality of life in survivors of childhood cancer after megatherapy with autologous bone marrow rescue. *Pediatr Hematol Oncol* **12**: 29–36

Kaplan SH, Barlow S, Spetter D, Sullivan L, Khan A and Grand R (1995) Assessing functional status and health-related quality of life among school aged children: reliability and validity of a new self-report measure (abstract). *Qual Life Res* **4**: 444

Karnofsky DA and Burchenal J (1949) The clinical evaluation of chemotherapeutics in cancer. In *Evaluation of Chemotherapeutic Agents*, MacLeod CM. (ed.), pp. 191–205. Columbia University Press: New York

Kelaghan J, Myers MH, Mulvihill JJ, Byrne J, Connelly RR, Austin DF, Strong LC, Meigs JW, Latourette HB and Holmes GF (1988) Educational achievement of long-term survivors of childhood and adolescent cancer. *Med Pediatr Oncol* **16**: 320–326

Kiltie EA and Rao Gattamaneni H (1995) Survival and quality of life of paediatric intracranial germ cell tumour patients treated at the Christie Hospital, 1972–1993. *Med Pediatr Oncol* **25**: 450–456

Koocher GP (1981) *The Damocles Syndrome*. McGraw Hill: New York

Landis RJ and Koch GG (1977) The measurement of observer agreement for categorical data. *Biometrics* **33**: 159–174

Lansky SB, List MA, Lansky LL, Ritter-Sterr C and Miller DR (1987) The measurement of performance in childhood cancer patients. *Cancer* **60**: 1651–1656

Larcombe IJ, Walker J, Charlton A, Meller S, Morris Jones P and Mott MG (1990) Impact of childhood cancer on return to normal schooling. *Br Med J* **301**: 169–171

Livesey EA, Hindmarsh PC, Brook CG, Whitton AC, Bloom HJ, Tobias JS, Godlee JN and Britton J (1990) Endocrine disorders following treatment of childhood brain tumours. *Br J Cancer* **61**: 622–625

Mancini AF, Rosito P, Canino R, Calzetti G, Di Caro A, Salmi S, Bonsi S, Marchi N, Paolucci G and Missiroli G (1989) School-related behaviour in children with cancer. *Pediatr Hematol Oncolol* **6**: 145–154

Mulhern RK, Heideman RL, Khatib ZA, Kovnar EH, Sanford RA and Kun LE (1994) Quality of survival among children treated for brain stem glioma. *Pediatr Neurosurg* **20**: 226–232

Mulhern RK, Wasserman AL, Friedman AG and Fairclough D (1989) Social competence and behavioral adjustment of children who are long-term survivors of cancer. *Pediatrics* **83**: 18–25

Pantel RH and Lewis CC (1987) Measuring the impact of medical care on children. *J Chronic Dis* **40** (suppl. 1): 99s–108s

Radcliffe J, Packer RJ, Atkins TE, Bunin GR, Schut L, Goldwein JW and Sutton LN (1992) Three- and four-year cognitive outcome in children with noncortical brain tumors treated with whole brain radiotherapy. *Ann Neurol* **32**: 551–554

Saigal S, Rosenbaum P, Stoskopf B, Hault L, Furlong W, Feeny D, Burrows and Torrance G (1994) Comprehensive assessment of the health status of extremely low birthweight children at eight years of age. *J Pediatr* **125**: 411–417

SIOP (1995) Guidelines for School/Education. *Med Pediatr Oncol* **25**: 429–430

Slavc I, Salchegger C, Hauer C, Urban C, Oberbauer R, Pakisch B, Ebner F, Schwinger W, Mokry M and Ranner G (1994) Follow-up and quality of survival of 67 consecutive children with CNS tumors. *Child's Nervous System* **10**: 433–443

Stiller CA (1992) Aetiology and epidemiology of childhood cancer. In *Paediatric Oncology, Clinical Practise and Controversies*, Plowman PN and Pinkerton CR. (eds), pp. 1–24. Chapman and Hall: London

Streiner DL and Norman GR (1995) *Health Measurement Scales. A Practical Guide to their Development and Use*. Oxford University Press: Oxford

Torrance GW, Boyle MH and Horwood SP (1982) Application of multi-attribute utility theory to measure social preferences for health states. *Operations Research* **30**: 1043–1069

Torrance GW, Zhang Y and Feeny D (1992) *Multi-attribute Preference Functions for a Comprehensive Health Status Classification System*. Working Paper No. 92–18, McMaster University, Centre for Health Economics and policy analysis: Hamilton (Ontario)

Torrance GW, Furlong W, Feeny D and Boyle M (1995) Multiattribute preference – Health Utility Index. *Pharmacoeconomics* **7**: 503–520

United Kingdom Children's Cancer Study Group (1992) PNET Study 111. University of Leicester

© Cancer Research Campaign 1997

Ware JE, Brook RH and Davies AR (1981) Choosing measures of health status for individuals in general populations. *Am J Public Health* **71**: 620–625
WHOQUOL Group (1993) *Measuring Quality of Life: The Development of the World Health Organisation Quality of Life Instrument (WHOQUOL)*. WHO: Geneva

### APPENDIX 1: QUESTIONS ON SELF-ESTEEM AND CONFIDENCE FOR THE FUTURE

### Self-esteem

Which one of the following describes how you see yourself?
a Excellent, as if on top of the world
b Very good, but a few problems
c Alright
d Not good, lots of problems
e Disastrous, could not be worse

### Confidence for the future

Which one of the following best describes the way you see your future?
a Extremely confident and positive
b Confident but occasional doubts
c Gloomy with occasional hope
d Very gloomy and dark with no hope

© Cancer Research Campaign 1997